# Blood Utilization Trends in Obstetrics and Gynecology: A Seven-Year Retrospective Study in a Teaching Hospital in Sikkim, India

**DOI:** 10.7759/cureus.45293

**Published:** 2023-09-15

**Authors:** Akanksha Sharma, Dhruva K Sharma, Supratim Datta

**Affiliations:** 1 Cardiology, Yatharth Super Specialty Hospitals, Greater Noida, IND; 2 Pharmacology and Therapeutics, Sikkim Manipal Institute of Medical Sciences, Sikkim Manipal University, Gangtok, IND

**Keywords:** cross-matching, obstetrics and gynecology, transfusion practices, blood center, blood components, whole blood, blood utilization indices

## Abstract

Introduction: Unutilized cross-matched blood due to excess cross-match requisitions results in unnecessary wastage of inventory, time, labor, and financial resources. This retrospective cross-sectional study aims to assess the blood utilization practices in obstetrics/gynecology (OB/GYN) over a period of seven years with respect to “blood utilization indices” and standard recommendations.

Material and methods: Cross-match requisitions from the OB/GYN Department over a period of seven years (2012-2018) were selected and included in the study using a suitable sampling technique. Patient details were retrieved from the Hospital Information System (HIS) database. The preoperative crossmatch requisitions and blood utilization data were recorded. “Blood utilization indices” and whole blood/component utilization patterns were analyzed.

Results: A total of 894 units of blood were cross-matched for 523 patients included in the study. A total of 305 of these patients were transfused with 445 units. During the initial phase of the study (2012-2014), the average cross-match-to-transfusion ratio (CTR, 6.6), transfusion probability (12.3), transfusion index (0.23), and component utilization (4%) were in marked deviation from recommended “blood utilization indices.” This was in contrast with the later phase of the study (2015-2018) wherein the average CTR (1.5), transfusion probability (69.3), transfusion index (1.3), and component utilization (91.8%) were compliant with recommended “blood utilization indices.”

Conclusion: A progressive improvement in blood utilization practices was observed in the OB/GYN Department during the study period. Awareness campaigns have contributed to the implementation of rational and judicious blood transfusion practices in our center.

## Introduction

Blood/blood products are one of the most precious lifesaving drugs in terms of potential benefit to recipients. Conversely, they are also considered to be one of the most dangerous drugs in terms of their potential to cause life-threatening adverse transfusion reactions [1]. In order to maximize benefits and minimize harm to patients, it is imperative to devise a multi-disciplinary, evidence-based, patient-centric approach that rationalizes the use of this valuable and limited but potentially harmful resource [2]. Safe and efficient blood transfusion services that are adherent to standard operating procedures are integral to the optimal functioning of healthcare delivery systems. Appropriate management of blood center resources ensures real-time availability of blood products and promotes service efficiency. Implementation of a pragmatic operational system that emphasizes on minimization of wastage is critical in achieving this objective.

According to the standard operating procedure, once blood centers receive requisitions for a specific number of blood units, the requisite number of units are reserved for the concerned department after processing routine cross-match. Once blood is cross-matched, the units remain unavailable for other patients. Consequently, excess cross-match requisitions beyond actual requirements may result in an inapparent blood scarcity, more so in facilities where blood is in short supply. Moreover, blood units cross-matched repeatedly without being issued sometimes end up being discarded following expiry. Another unwanted outcome of excess ordering of blood is the imposition of additional financial strain on patients [3]. The availability of life-saving blood in vulnerable groups of patients, such as women and children, is also at risk of being compromised. Cross-match requisitions for elective and emergency procedures from obstetrics/gynecology (OB/GYN) departments are found to be relatively higher and beyond that required, as compared to those of other surgical departments [4]. Despite clearly enunciated and recommended blood transfusion guidelines [5], there is a paucity of knowledge pertaining to the judicious use of blood or blood components, more so among clinicians in OB/GYN departments [6].

Sikkim is a remote, landlocked, hilly state located in the northeast region of India. Two tertiary care hospitals located in the East District of Sikkim cater to the healthcare requirements of the Sikkimese population. Both hospitals have a huge demand for blood/blood products. The remote location and sparse population make it challenging to recruit adequate healthy blood donors, despite frequent blood donation camps. It thus becomes essential to ensure an adequate supply of blood and blood products commensurate with demand by ensuring rational utilization and avoiding irrational blood transfusion practices [7]. This translates to providing the right blood or blood products, in the right quantity, to the right patient and at the right time.

The “component processing services” in our blood center were initiated in the year 2012, prior to which only whole blood (WB) was available. There has been no assessment of blood utilization practices since the introduction of blood component facilities. This study was thus conducted with the objective of evaluating the annual blood ordering practices and analyzing the blood utilization indices in the OB/GYN Department, following the introduction of “component processing services.”

## Materials and methods

Study design and setting

This retrospective cross-sectional analytical study was conducted in the Blood Center, a constituent unit of the Department of Pathology in a teaching hospital in Sikkim.

Ethics

Due approval of the Institutional Research Protocol Evaluation Committee (Registration No. IRPEC/398/19-014) and Institutional Ethics Committee (Registration No. IEC/522/19-43) was obtained prior to the initiation of the study.

Sample size and sampling technique

The study included data ranging from the 1st of January 2012 to the 31st of December 2018 (a total period of seven years). Cross-match requisition forms received from the OB/GYN Department were included in the study using simple random sampling as per the estimated sample size. Sample size calculation was done using OpenEpi software. The calculated sample size was 462. Accounting for 10% incomplete data, a minimum sample of 508 was estimated. The calculated sample for each year (estimated sample divided by seven years) was selected using simple random sampling generated through a random sequence producer. The demographic and relevant clinical data of patients from the OB/GYN Department for whom WB/blood product requisitions were made was obtained from the crossmatch requisition forms, registers, and the electronic Hospital Information System (HIS) database.

Data analysis

The data thus obtained were entered into a Microsoft Excel spreadsheet, and statistical analysis was done using Statistical Product and Service Solutions (SPSS), version 27.0 (IBM SPSS Statistics for Windows, Armonk, NY). Data obtained from patients suffering from known bleeding disorders were excluded from the study. Data analysis was based on the following salient parameters: age of the patient, type of blood (component or WB), transfusion units (multiple or single), indication for transfusion, and number of units cross-matched versus number of units transfused. Lastly, the study computed and scrutinized "blood utilization indices" in accordance with the established guidelines [3,7,8].

Blood utilization indices

i. Cross-match-to-transfusion ratio (CTR) = Number of units cross-matched/number of units transfused.

A ratio of 2.5 and below is considered indicative of significant blood usage [3,4,7,8].

ii. Transfusion probability (%T) = Number of patients transfused/number of patients cross-matched ×100.

A value of 30% and above is considered to indicate efficient blood usage [3,8].

iii. Transfusion index (TI) = Number of units transfused/number of patients cross-matched.

The average number of units used per patient cross-matched is indicated by the TI.

A value >0.5 is considered indicative of significant blood utilization [3,7,8].

The "initial phase" spans from 2012 to 2014 and represents the initial three years following the implementation of blood component services. During this period, the hospital's blood transfusion committee actively engaged in educational efforts, conducting scientific sessions to inform clinicians about the availability and benefits of using blood components, instead of WB transfusions. Essentially, this phase serves as a "training phase" where clinicians learn about and adapt to the new approach.

On the other hand, the "later phase" covers the remaining study period from 2015 to 2018. This phase can be considered a "consolidation phase" because, by this time, clinicians had become well-informed about the availability and advantages of blood component services. The educational efforts from the initial phase had taken effect, and clinicians had integrated this knowledge into their practices. Therefore, this later phase reflects a stage where the utilization of blood components had likely become more established and routine.

By dividing the study into these two phases, we aimed to assess how knowledge dissemination and adoption of blood component services evolved within the hospital, providing valuable insights into the impact of these services on clinical decision-making and patient care.

## Results

**Figure 1:**
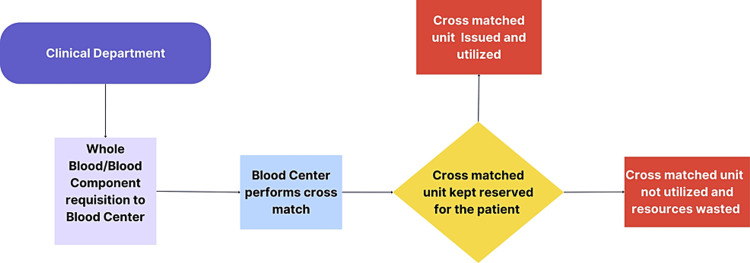
Blood supply management workflow: cross-match requisition and implications

Cross-match requisition data of 523 patients, during the study period (2012-2018), were collected and analyzed following the standard operating procedures (Fig. 1). The mean age of the study population was 28.2 years (SD: 6.0). There was a demand of 894 units of WB/blood components to be cross-matched against 523 selected crossmatch requisitions. A total of 305 of these patients for whom cross-match requisition was made were transfused with 445 units. Among the total number of patients transfused, 99 patients received single unit transfusion.

Blood transfusion indices

%T

%T was assessed during the study period spanning from 2012 to 2018. In the initial phase, %T was found to be 10.6% in 2012, dropping to 8.1% in 2013, and increasing to 18.4% in 2014. It is worth noting that, during this initial phase, %T consistently remained below the recommended cutoff of 30%. However, a notable shift was observed in the later phase (2015-2018), where the average %T significantly increased to 69.3%, which is markedly higher than the average of 12.3% for the initial period.

TI

Analysis of the TI revealed noteworthy variations between the two study phases. In the initial phase, the TI was calculated to be 0.23, a value markedly lower than the desired threshold of >0.5, indicating a suboptimal transfusion practice. Conversely, during the later phase (2015-2018), the average TI improved substantially, reaching 1.3, thereby aligning more closely with the desired value.

CTR

The CTR data unveiled a clear disparity between the two study phases. In the initial phase, the CTR was notably high at 6.6, signifying a considerable excess cross-match ordering practices. Further examination revealed specific yearly CTR values of 6.1 (2012), 9.4 (2013), and 4.3 (2014), all well above the recommended standard of <2.5. However, in the later phase, the average CTR improved substantially, achieving a value of 1.5 (Table 1), which aligns more closely with the standard.

**Table 1:** Cross-match data and blood transfusion indices (2012-2018)

Year	No. of units cross-match requested (A)	No. of units transfused (B)	No. of patients cross-matched (C)	No. of patients transfused (D)	Cross-matched units not transfused (E)	Cross-match-to-transfusion ratio (CTR) A/B	Transfusion Probability (% transfused) D/C	Transfusion index (TI) B/C	Excess cross-match done in % E/A x 100
2012	92	15	75	8	77	6.1	10.6	0.2	83.6
2013	141	15	74	6	126	9.4	8.1	0.2	89.3
2014	99	23	76	14	76	4.3	18.4	0.3	76.7
2015	106	54	74	39	52	1.9	52.7	0.7	49.0
2016	144	130	76	70	14	1.1	92.1	1.7	09.7
2017	148	82	74	39	66	1.8	52.7	1.1	44.5
2018	164	126	74	59	38	1.3	79.7	1.7	23.1
Overall 2012-2018	894	445	523	305	449	1.6	44.9	0.8	53.7

**Figure 2:**
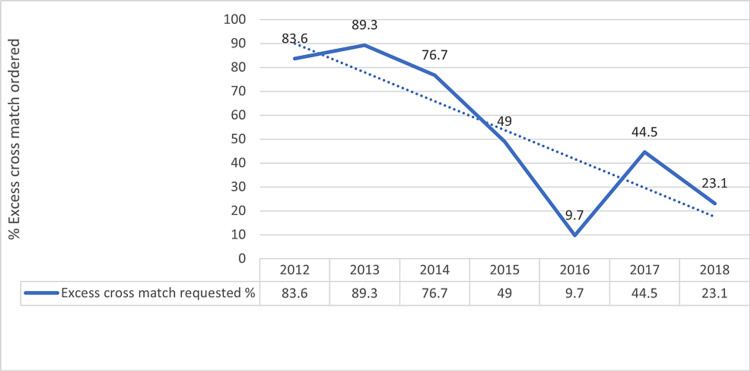
Trend of excess cross-match (2012-2018)

A trend map (Fig. 2) was generated to visually represent the correlation between transfusion and cross-match requests throughout the study period. This graphical depiction illustrates a progressive decline in the number of excess cross-match requests over the years, offering a visual insight into the evolving transfusion practices.

**Figure 3:**
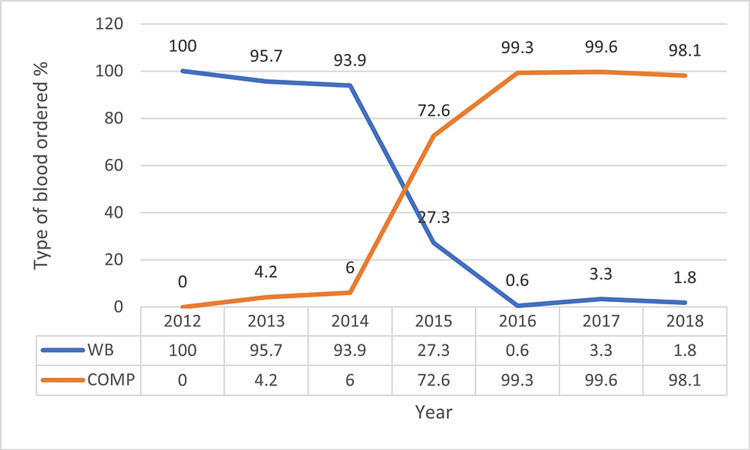
Trend of requisition of whole blood and component (2012-2018)

Requisitions for WB were most frequent between the years 2012-2014 (average: 96.5%) as compared to the average component requisition of 3.4%. Between the years 2015 and 2018, the average component requisition substantially increased to 91.6%, whereas the average WB requisition lowered to 8.2% (Table 2). Fig. 3 depicts the changing trends in the type of blood requested by the OB/GYN Department during the study period.

**Table 2:** Comparative utilization of whole blood and blood components (2012-2018) Figures in parentheses indicate percentages. WB = Whole Blood, COMP = Component

Year (n=units requested)	Year (n=units issued)	Blood type requested	Blood type issued
2012 (n=92)	2012 (n=15)	WB: 92 (100)	WB: 9 (60)
		COMP: --	COMP: 6 (40)
2013 (n=141)	2013 (n=15)	WB: 135 (95.7)	WB: 7 (46.6)
		COMP: 6 (4.2)	COMP: 8 (53.3)
2014 (n=99)	2014 (n=23)	WB: 93 (93.9)	WB: 2 (8.6)
		COMP: 6 (6)	COMP: 21(91.3)
2015 (n=106)	2015 (n=54)	WB: 29 (27.3)	WB: 4 (7.4)
		COMP: 77 (72.6)	COMP: 50 (92.5)
2016 (n=144)	2016 (n=130)	WB: 1 (0.6)	WB: --
		COMP: 143 (99.3)	COMP: 130 (100)
2017 (n=148)	2017 (n=82)	WB: 5 (3.3)	WB: --
		COMP: 143 (96.6)	COMP: 82 (100)
2018 (n=164)	2018 (n=126)	WB: 3 (1.8)	WB: --
		COMP: 161 (98.1)	COMP: 126 (100)
Overall 2012-2018 (n=894)	Overall 2012-2018 (n=445)	WB: 249 (27.8)	WB: 22 (4.9)
		COMP: 645 (72.1)	COMP: 423 (95.1)

The most common indication for blood requisition from the OB/GYN Department was for antenatal care (86%), which was scheduled for vaginal delivery or lower segment cesarean section (LSCS). Other indications included incomplete abortion, postpartum hemorrhage (PPH), ectopic pregnancy (ruptured/unruptured), and hysterectomy amongst various others (Table 3).

**Table 3:** Indications for blood requisition from OB/GYN Department (2012-2018) ANC/LSCS = Antenatal care/lower segment caesarean section, PPH = Post-partum hemorrhage, MTP = Medical termination of pregnancy, DUB = Dysfunctional uterine bleeding, DNC = Dilatation and curettage

Clinical diagnosis	Number (n=523)	Percentage (%)
ANC/LSCS	451	86.2
Incomplete abortion	16	3.1
PPH	11	2.1
Ectopic pregnancy	8	1.5
Hysterectomy	7	1.3
Menorrhagia	4	0.8
Missed abortion	4	0.8
Suction evacuation	4	0.8
Bartholin cyst	3	0.6
MTP	3	0.6
Fibroid Uterus	3	0.6
Ovarian cyst	3	0.6
Uterovaginal prolapse	2	0.4
DUB	1	0.2
Retained placenta	1	0.2
DNC	1	0.2
Threatened abortion	1	0.2

## Discussion

There has been a trend of over-ordering of blood due to an over-assumption of intraoperative blood loss. The decision to transfuse blood is frequently based on a subjective assessment of a particular procedure. The need to develop or follow a blood order and/or transfusion policy is frequently overlooked. This trend is more common in developing countries where excess ordering of blood has been observed in 40%-70% of all transfusions [8]. This trend is evident in the findings during the initial phase of our study (2012-2014) where the average excess cross-match was 83.2%. However, excess cross-match in the later phase (2015-2018) reduced to 31.5%. The CTR, first described by Zewdie et al. [9], is a blood utilization index, which evaluates the efficiency of transfusion policy. The CTR is a benchmark for clinical practice that indicates significant blood usage and is expected to be 2.5 or lower [3,8,10,11]. It is advised that at least 33% of the cross-matched units should be transfused. The probability of blood transfusion (i.e., %T) is expressed as a percentage and is another index to assess the appropriateness of blood transfusion. Transfusion probability was first described by Meadet al. and Khoshrang et al. [12,13] in 1980 as one of the indicators of judicious use of blood. A value of >50% is desirable and indicates a considerable requirement of blood [3], and a value of at least 30% indicates efficient blood usage. %T for the initial phase of the study averaged 12.3%, which is comparable to the results of another study [9], where %T for gynecological disorders was 16.33% and 6.45% for obstetrics. These findings were far below the suggested threshold values recommended by international guidelines. This suggests that, on average, only 10%-15% of the patients that were cross‑matched required blood transfusion. However, during the later phase of our study, the transfusion probability significantly improved, averaging 69.3%, which is comparable to the findings of a study (%T=41.26%) conducted by Raghuwanshi et al. and Zewdie et al. [3,9] in an OB/GYN department. TI is another criterion that reflects the average number of units used per patient. Values >0.5 are a good indicator of the quality of blood utilization services. The TI calculated for the entire study period was 0.8. Considering the initial study period, the TI value was 0.23, which is much lower than the recommended cutoff value. These findings suggest that the blood ordering practices and blood utilization indices during the initial study period were not in adherence to recommendations. Another study reported a similar transfusion index of 0.27 for gynecology and 0.06 for obstetrics [9]. A considerable improvement in the pattern of blood use was noted, in the later phase of the study period, during which the average TI improved to 1.3. Of the 523 patients included in our study, 451 were cases of antenatal care (ANC) posted for normal vaginal delivery/LSCS. A cross-tabulation between indications for transfusion and the number of units transfused revealed that 181 of 451 patients of ANC (40.1%) did not require transfusion. In contrast, a study conducted in Nigeria reported that 90.9% of ANC cases did not require any transfusion [14]. The fact that the transfusion rate in ANC patients at our hospital was relatively high could be attributed to individual healthcare providers' blood transfusion preferences. To minimize needless excess preoperative cross-matching, hospitals all over the world have adopted a "Maximum Surgical Blood Order Schedule (MSBOS)" as a quality indicator. MSBOS, which is also known as Mead's criteria, calculated as (1.5 × TI), serves as a guideline for the most often performed elective procedures, advising the number of blood units that should be cross-matched beforehand. This eventually leads to a decrease in the number of unnecessary preoperative crossmatch requests. It has been universally observed that MSBOS has substantially reduced unnecessary cross-match, brought down direct or indirect costs, and has proven to be the most effective means for the reduction of blood wastage by expiry of blood units [8,11,15,16]. Implementation of such a blood ordering policy may not be possible without the cooperation and mutual understanding of all stakeholders involved, such as surgeons, anesthetists, hematologists, hospital administration, and blood center services. Transfusion needs are, however, individualized, and there is no accurate method to predict blood loss or intraoperative variations.

According to WHO blood safety guidelines, WB transfusions should be avoided as a treatment for anemia [5] and should not be used routinely to treat isolated blood component deficiencies (e.g., anemia in non-bleeding hospitalized patients) [17]. The use of WB is recommended for selective conditions, such as resuscitation of patients experiencing massive blood loss/hemorrhagic shock. Despite the initiation of component processing facilities in our blood center in the year 2012, most of the blood requisitions from OB/GYN during the initial phase were for WB (average of 96.5%). A study conducted by Gupte [18] reported that 58.5% of transfusions in OB/GYN patients were in the form of WB [18]. Separation of blood into components has its own advantages, which include facilitating each donation to benefit three patients instead of one and minimizing risks associated with multiple transfusions. Way back in 1987, Hogman et al. [19] recommended that WB should be avoided in the management of bleeding during surgery or postoperative bleeding. Nevertheless, many anesthetists and surgeons in India still insist on transfusing WB to control such bleeding [18]. Following the initiation of a component facility in our blood center, the hospital's transfusion committee and the blood center started organizing frequent sensitization programs and scientific sessions aimed at enhancing awareness amongst clinicians pertaining to the use of components and rational use of blood/blood products. The concerted efforts to promote awareness amongst clinicians eventually translated into a tangible improvement in blood utilization indices and optimum use of components during the later phase of the study period. This suggests that awareness campaigns are likely to bring a change in practices once the message has percolated across a major segment of clinician stakeholders.

Another criterion for the judicious use of blood is avoiding over-transfusion in patients. For stable, normovolemic patients who are not actively bleeding and are not in an operating room, one-unit transfusions are typically regarded as safe and appropriate [20,21]. Transfusion of further units merely increases the hazards without offering any additional advantages. In our study, many of the patients (32.4%) received single-unit blood transfusions. In >80% of Women with hemodynamically stable postpartum anemia, a single-unit approach avoids a second unit of packed red blood cells without significantly increasing morbidity [20,21]. Hemoglobin threshold should not be the sole criterion for determining transfusion requirement. A pragmatic assessment of the physiological and pathological status of the patient should also be considered determinants for transfusion. A two-unit transfusion enhances the risk of nosocomial infection and other long-term morbidities, such as transfusion-associated circulatory overload (TACO) [22].

Frequent institutional transfusion audits can provide the foundation for implementing an institutional blood utilization policy, based on MSBOS. MSBOS is designed to order enough blood for 85%-90% of patients for each surgical procedure and would help in ensuring adequate responsiveness of the transfusion services according to the changing needs of the hospital. This would eventually lead to a decrease in the wastage of resources, workload, and financial burden to the patients and contribute to improving the efficiency of transfusion services. This study underlines the need for a comprehensive MSBOS guideline document that clearly defines the criteria pertaining to cross-match requirements.

The study, being a student project, was confined to the OB/GYN Department considering time constraints. There is a future scope of conducting a more comprehensive assessment of transfusion practices that includes all surgical departments and specific surgical procedures to formulate a hospital-based MSBOS guideline.

## Conclusions

Maintaining an adequate stock of blood and blood products requires healthy blood donors and frequent blood donation camps. Since Sikkim is sparsely populated, it is often difficult to identify an adequate number of voluntary blood donors. It, thus, becomes imperative to avoid any preventable wastage of blood by improving blood utilization practices. This study indicates that awareness campaigns are crucial in the implementation of rational and judicious blood transfusion practices. It is recommended that blood center audits be carried out at least once a year and formulate/update hospital MSBOS policy.
